# The Normal and Pathological Circulation in the Central Nervous System (Myel-Encephalon)

**Published:** 1897-11

**Authors:** 


The Normal and Pathological Circulation in the Central Nervous System (Myel-Encephalon). Original Studies by William Browning, Ph. B., M. D., Attending Neurologist to the Kings County Hospital and Consulting to the St. Christopher's Hospital for Babies, etc., etc. Small 8vo, pp. 171. Price, $1.50. Philadelphia: J. B. Lippincott Co. 1897.

The author has presented in an original and concise manner facts concerning the normal and pathological circulation of the central nervous system. Many of the chapters,—papers written by the author during the past ten years,—are here collected, systematised and made a part of a very interesting and instructive treatise. These serve as a basis and further observations and additions by the author and by others give a very clear idea of the encranial circulation and its disturbances. The first six chapters are anatomical and experimental; the remaining twelve take up the clinical and pathological aspects. The veins of the brain in the monkey have thus far received little attention and the author has gone into this question somewhat at length and has illustrated his research with two plates.

Regarding the value of lumbar puncture, the author says, page 59:

As a diagnostic means—*e. g.*, in suspected meningeal hemorrhage, it is valuable and as an index of pressure it may also be worth noting.

It is worth further trial: (*a*) as a passing relief in brain tumors not complicated with hydrocephalus; (*b*) as a substitute for trephining in progressive dementia; (*c*) in certain spinal troubles; (*d*) and possibly as a means of applying medication directly to the spinal meninges.

Time alone can tell whether these hopes will all be realised, but doubtless its future field of application will grow narrower as reports come in.

The closing chapters are devoted to clinical cases affecting the cerebral circulation and vessels and are well presented. It is, we believe, the first treatise devoted to this subject and is worthy of perusal and commendation. It is printed on good paper, excellently bound and a good example of the bookmaker's art.

